# Artificial intelligence enabled automated diagnosis and grading of ulcerative colitis endoscopy images

**DOI:** 10.1038/s41598-022-06726-2

**Published:** 2022-02-17

**Authors:** Reed T. Sutton, Osmar R. Zai͏̈ane, Randolph Goebel, Daniel C. Baumgart

**Affiliations:** 1 Division of Gastroenterology, University of Alberta, 130 University Campus, Edmonton, AB T6G 2X8 Canada; 2 Department of Computing Science, University of Alberta, Edmonton, AB Canada; 3 Alberta Machine Intelligence Institute, University of Alberta, Edmonton, AB Canada

**Keywords:** Inflammatory bowel disease, Ulcerative colitis, Computational science, Computational models, Learning algorithms, Network models

## Abstract

Endoscopic evaluation to reliably grade disease activity, detect complications including cancer and verification of mucosal healing are paramount in the care of patients with ulcerative colitis (UC); but this evaluation is hampered by substantial intra- and interobserver variability. Recently, artificial intelligence methodologies have been proposed to facilitate more objective, reproducible endoscopic assessment. In a first step, we compared how well several deep learning convolutional neural network architectures (CNNs) applied to a diverse subset of 8000 labeled endoscopic still images derived from HyperKvasir, the largest multi-class image and video dataset from the gastrointestinal tract available today. The HyperKvasir dataset includes 110,079 images and 374 videos and could (1) accurately distinguish UC from non-UC pathologies, and (2) inform the Mayo score of endoscopic disease severity. We grouped 851 UC images labeled with a Mayo score of 0–3, into an inactive/mild (236) and moderate/severe (604) dichotomy. Weights were initialized with ImageNet, and Grid Search was used to identify the best hyperparameters using fivefold cross-validation. The best accuracy (87.50%) and Area Under the Curve (AUC) (0.90) was achieved using the DenseNet121 architecture, compared to 72.02% and 0.50 by predicting the majority class (‘no skill’ model). Finally, we used Gradient-weighted Class Activation Maps (Grad-CAM) to improve visual interpretation of the model and take an explainable artificial intelligence approach (XAI).

## Introduction

Ulcerative colitis and Crohn’s disease, together referred to as inflammatory bowel disease(s) (IBD), are two chronic systemic inflammatory disorders^1,2^. They result from an inappropriate immune response towards the commensal microbiota in genetically susceptible individuals^3^. Ulcerative colitis cannot be cured, requires lifelong medical therapy^4^, and can progress from repeated flare-ups to complete digestive failure^5^.

Endoscopy is paramount in establishing the initial diagnosis, evaluating disease extent or disease activity, assessing disease complications, providing cancer surveillance and can establish a hard endpoint in clinical trials investigating new treatments^6,7^. Therapeutic strategy has evolved towards seeking combined hard endpoints (such as clinical and endoscopic remission)^8,9^. Mucosal healing has been associated with favorable long-term outcomes^10,11^.

However, endoscopic scoring systems for ulcerative colitis are heterogeneous and subjective, with significant inter- and intra-observer variability; *and are still not routinely used in clinical practice*^12^. Even in randomized controlled trials, there is great variation in their application and interpretation^13^. Standardization of scoring through unbiased remote central reading is an ideal solution, but not feasible in daily clinical practice^14^.

Machine learning (ML), computer vision (CV) and other algorithmic methodologies commonly referred to as artificial intelligence (AI) techniques have shown promise in mostly classic radiologic diagnostic imaging. The available literature suggests that AI models are capable of being as accurate or superior to human experts at certain medical tasks^15–17^.

But application of AI in the context of inflammatory bowel diseases is in the very early stages^18^. Preliminary evidence suggests that convolution neural networks (CNN) may be useful to classify severity of ulcerative colitis on endoscopic images^19–21^. However, more data and validation are required to inform analysis approaches and algorithm selection. Here, we investigate the ability of deep learning^22^ algorithms to distinguish ulcerative colitis from other classes of intestinal disorders and grade the endoscopic severity of ulcerative colitis using a weakly supervised approach^23^.

## Methods

### Dataset

Kvasir is a multi-class dataset from Bærum Hospital in Vestre Viken Health Trust (Norway), collected from 2010 to 2014^24^. Kvasir (v2) contains 8000 endoscopic images labelled with eight distinct classes, with approximately 1000 images per class, including ulcerative colitis. The images are assigned only image-level labels, provided by at least one experienced endoscopist as well as medical trainees (minimum of 3 reviewers per label). The images are independent, with only one image per patient.

Standard endoscopy equipment was used. HyperKvasir is an extension of the Kvasir dataset, collected from the same Bærum Hospital from 2008 to 2016, containing 110,079 images, 10,662 of which are labelled with 23 classes of findings^25^. Pathological findings in particular accounted for 12 of 23 classes, which are aggregated and summarized in Table 1. They can be broadly grouped into Barret’s esophagus and esophagitis in the upper GI tract, and polyps, ulcerative colitis, and hemorrhoids in the lower GI tract.

**Table 1:** Number of labelled images for each pathological finding contained in the *HyperKvasir* dataset.

GI tract segment	Pathological finding	# Images
Upper	Barret’s (2 classes)	94
Upper	Esophagitis (2 classes)	663
Lower	Polyps	1028
Lower	Ulcerative colitis (6 classes)	851
Lower	Hemorrhoids	6

Importantly, the dataset includes 851 ulcerative colitis images which are labelled and graded using the Mayo endoscopic subscore^26,27^ by a minimum of one board certified gastroenterologist and one or more junior doctors or PhD students (total of 3 reviewers per image). The images are in JPEG format, with varying image resolutions, the most common being 576 × 768, 576 × 720, and 1072 × 1920. Table 2 shows the number of images available for each Mayo grade.

**Table 2:** Number of images for each ulcerative colitis Mayo score grade contained in the HyperKvasir dataset.

Mayo grade	Findings	# Images
Grade 0	Inactive, mucosa has normal vasculature	0
Grade 0–1*	–	35
Grade 1	Mild with erythema, decreased vascular pattern, mild friability	201
Grade 1–2*	–	11
Grade 2	Moderate with erythema, absent vascular pattern, mild friability, erosions	443
Grade 2–3*	–	28
Grade 3	Severe with spontaneous bleeding and ulcerations	133

*Ulcerative colitis images in HyperKvasir were classified with partial grades (not a common clinical practice) as they reported higher inter/intraobserver classification on this dataset.

The HyperKvasir study, including the HyperKvasir dataset available through the Center for Open Science we are using here, was approved by Norwegian Privacy Data Protection Authority, and exempted from patient consent because the data were fully anonymous. All metadata was removed, and all files renamed to randomly generated file names before the internal IT department at Bærum hospital exported the files from a central server. The study was exempted from approval from the Regional Committee for Medical and Health Research Ethics—Southeast Norway since the collection of the data did not interfere with the care given to the patient. Since the data is anonymous, the dataset is publicly shareable and complies with Norwegian and General Data Protection Regulation (GDPR) laws. Apart from this, the data has not been pre-processed or augmented in any way.

### Training objectives

**Figure 1:**
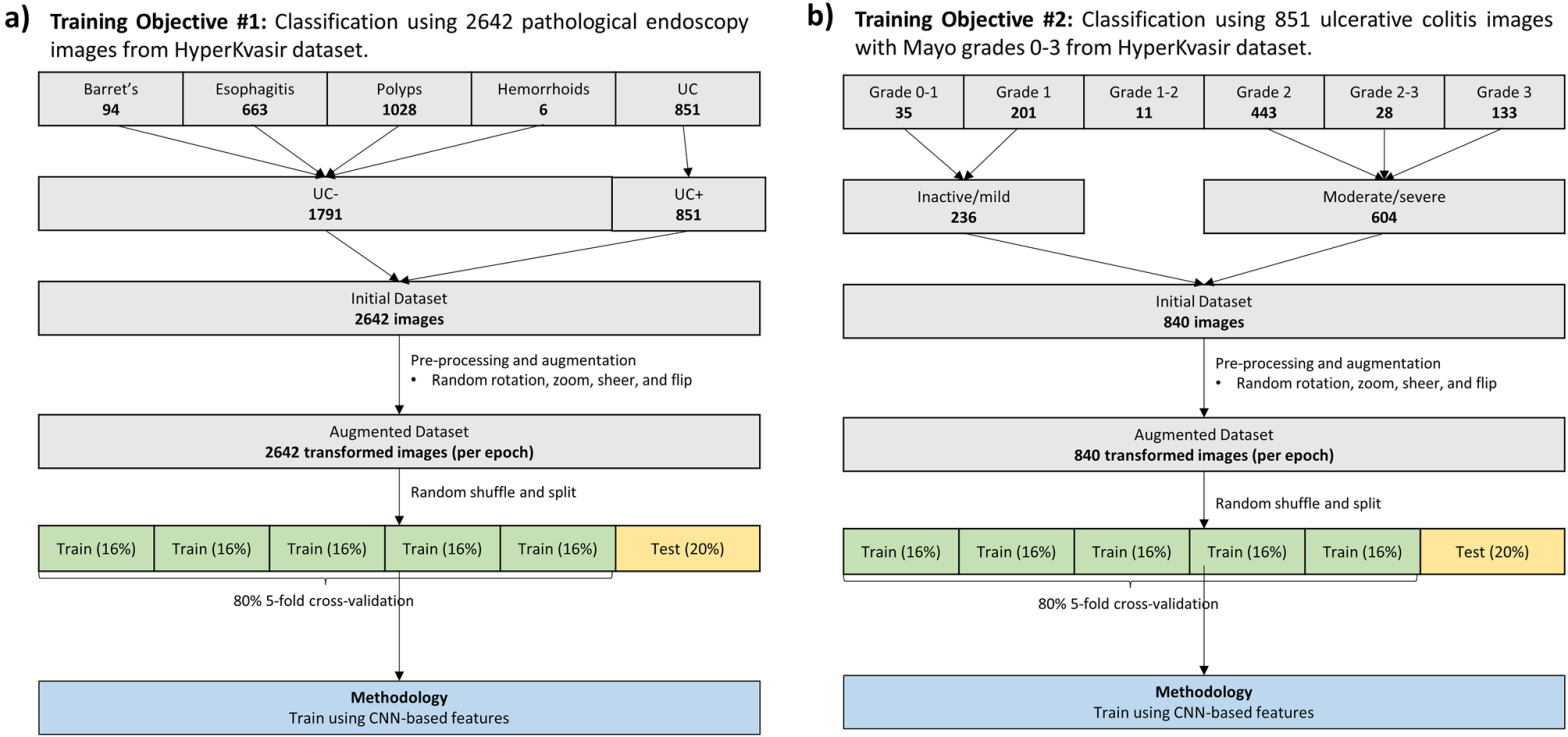
Methods (**a**) Overview of methods used to train diagnostic classification model of ulcerative colitis from multi-class endoscopic images on Kvasir datasets. (**b**) Overview of methods used to train diagnostic model for endoscopic grading of ulcerative colitis on HyperKvasir dataset.

Two binary classification tasks were formulated from the dataset:

Diagnosis: All pathological findings of ulcerative colitis were grouped along with all other classes of pathological findings in the dataset (Fig. 1a). The problem was formulated as a binary classification task to distinguish UC from non-UC pathology on endoscopic still images.

Grading: Evaluation of disease severity using endoscopic images of UC pathology. Mayo graded image labels were binned into Grades 0–1 and 2–3. (Fig. 1b) This grouping has been used in previous machine learning studies and for clinical trial endpoints^19^. Therefore, the task was to distinguish inactive/mild from moderate/severe UC.

### Data preprocessing

A filter was designed to remove the green picture-in-picture depicting the endoscope. The filter applied a uniform crop to all images, filling in the missing pixels with 0 values, turning them black.

Source images were then normalized to [− 1, 1] and downscaled to 299 × 299 resolution using bilinear resampling. Images underwent random transformations of rotation, zoom, sheer, vertical and horizontal flip, using a set seed. Image augmentation was only applied to training set images (not validation or test set), inside each fold of the fivefold cross-validation.

### Model generation

There are a growing variety of machine learning frameworks that could provide the foundation for our study. Our choices here acknowledge the current dominance of deep neural network methods, despite the emerging challenges of explainability (explainable artificial intelligence = XAI) and trust in practical clinical implementation^41^. Most of our choices use the most popular method for classifying images (convolutional neural networks), whose major differences lie in their depth of layering (50–160) and recorded dimensionality of annotated relationships amongst segments of images (up to 2048).

The following four different CNN architectures were tested on the Kvasir dataset:

- Pre-trained *InceptionV3*, a 159-layer CNN. The output of InceptionV3 in this configuration is a 2048-dimensional feature vector^28^.
- Pre-trained *ResNet50*, a Keras implementation of ResNet50, a 50-layer CNN which uses residual functions that reference previous layer inputs^29^.
- Pre-trained *VGG19*, a Keras implementation of VGG which is a 19 layer CNN developed by Visual Geometry Group^30^.
- Pre-trained *DenseNet121*, a Keras implementation of DenseNet with 121 layers^31^.

All pre-trained models were TensorFlow implementations initialized using ImageNet weights^32^.Training was performed end-to-end with no freezing of layers. All models performed a final classification step via a dense layer with one node. Sigmoid activation was used at this final dense layer, with binary cross entropy for the model’s loss function.

### Validation framework

For both classification tasks, the final dataset was randomly shuffled and split into training and validation sets in a 4:1 ratio, where 80% images were used for fivefold cross-validation and 20% unseen images were used for evaluating model performance. The best model from each fold were combined and used as the final model for prediction on the test set.

### Hyperparameters tuning

Hyperparameters were fine-tuned using Grid Search, where the search space included the following parameters: optimizers: Adam, Stochastic Gradient Descent (SGD), learning rate: 0.01, 0.001, 0.0001; momentum (for SGD): 0, 0.5, 0.9, 0.99. For all models, training phases consisted of 20 epochs with batch size of 32.

### Evaluation metrics

Models were evaluated using accuracy, recall, precision, and F1-scores. As a binary classification problem, confusion matrices and ROC curves were used to visualize model performance.

### Explainability analysis (XAI)

To provide visual explanation of what the models are learning, we chose the Gradient-weighted Class Activation Mapping (Grad-CAM) technique^33^. Grad-CAM produces a heatmap for each model output, showing which part(s) of the image the model is using to make predictions (produces the strongest activation). The heatmap is a course localization map produced by using gradient information flowing into the last convolutional neural network layer, to assign importance values to each neuron.

We also had an experienced gastroenterologist (D.C.B.) annotate and highlight the regions of interest in representative images to provide a comparison with the regions of interest generated by the heatmaps.

### Software implementation

Model building was performed and figures created was done using TensorFlow and Keras packages^32^ in Python 3.6.9, run on Google Colab (https://research.google.com/colaboratory/) notebook.

## Results

### Model performance

**Figure 2:**
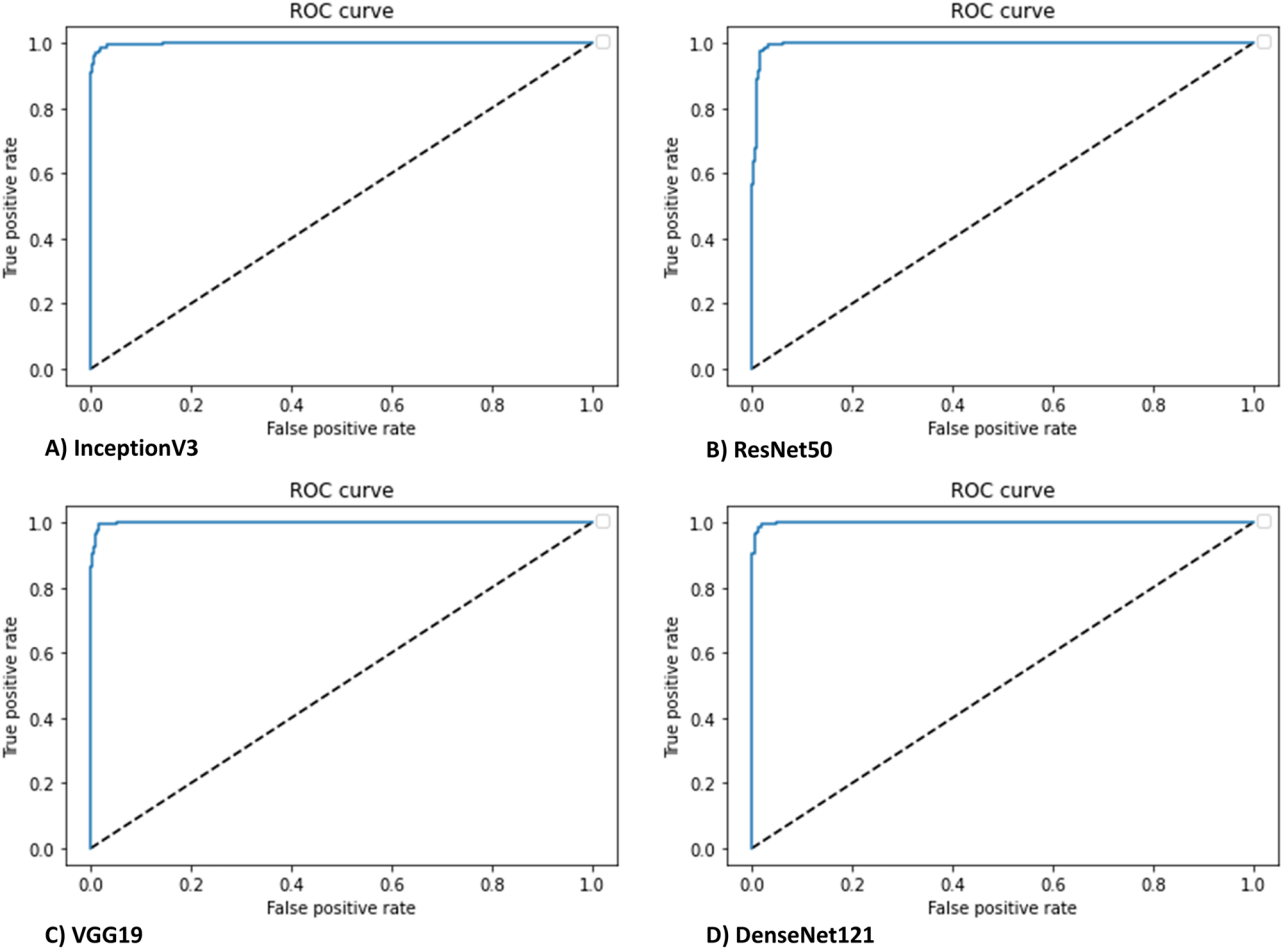
CNN discrimination between ulcerative colitis and non-ulcerative colitis pathologies had an area under the receiver operating curve (AUROC) of > 0.99 with all model architectures (InceptionV3, ResNet50, VGG19, DenseNet121. Dashed lines represent a nondiscriminatory AUROC (0.5).

In comparing ulcerative colitis with non-ulcerative colitis endoscopic pathologies, all four of our CNN models achieved very high predictive accuracy in all experiments. Table 3 shows the evaluation metrics performed on the test dataset for each model. The highest AUROC of 0.999 was achieved with DenseNet121, however this did not achieve statistical significance with respect to all other model architectures having extremely high AUROCs (Fig. 2).

**Table 3:** Evaluation metrics of candidate models for ulcerative colitis diagnosis (binary classification task #1).

Model	Metrics				
	*ACC*	*SEN*	*SPEC*	*F1*	*AUC*
InceptionV3	97.92	98.61	96.47	96.76	0.9978
ResNet50	98.11	98.33	97.65	97.08	0.9958
VGG19	98.49	98.61	98.24	97.66	0.9988
DenseNet121	98.30	98.89	97.06	97.35	0.9990
Majority class	67.86	100	0	48.64	0.5

**Figure 3:**
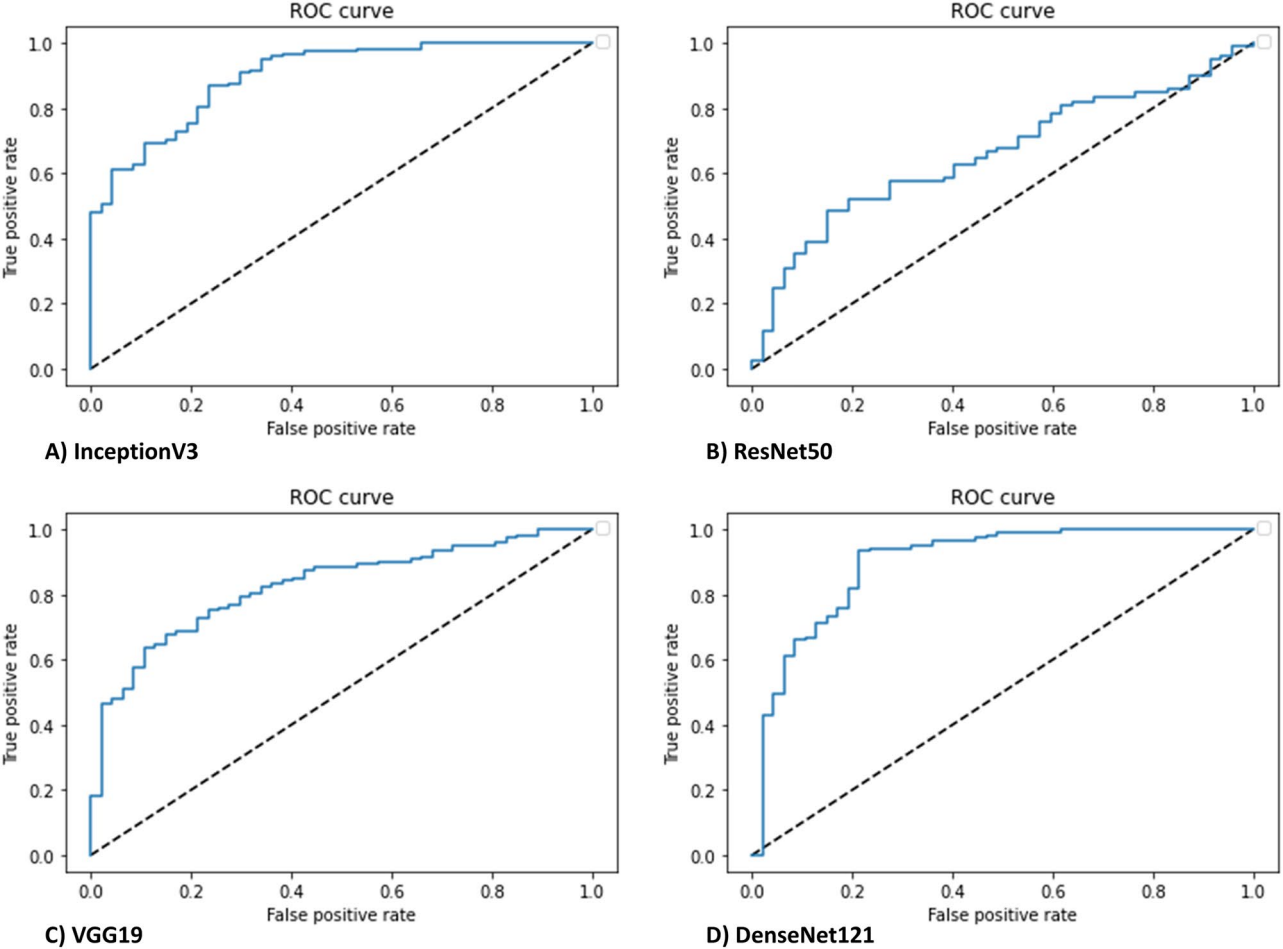
CNN discrimination between endoscopic remission (Mayo 0 or 1) from moderate to severe activity (Mayo 2 or 3) had an area under the receiver operating curve (AUROC) of 0.90 with InceptionV3 (**A**), 0.66 with ResNet50 (**B**), 0.83 with VGG19 (**C**), and 0.90 with DenseNet121 (**D**). Dashed lines represent a nondiscriminatory AUROC (0.5).

In comparing endoscopic remission (Mayo subscore of 0 or 1) with moderate to severe active disease (2 or 3), based on the US FDA definition^34^, all models achieved varying prediction accuracy. Table 4 shows the evaluation metrics performed on the test dataset for each model. The highest AUROC was achieved with DenseNet121, however this did not achieve statistical significance when compared to InceptionV3 results. On the other hand, the shallower CNNs (ResNet50, VGG19), were unable to achieve better accuracy than majority class prediction. AUROC curves are shown in Fig. 3 for the four different CNN architectures.

**Table 4:** Evaluation metrics of candidate models for ulcerative colitis disease activity prediction (binary classification task #2).

Model	Metrics				
	*ACC*	*SEN*	*SPEC*	*F1*	*AUC*
InceptionV3	84.52	68.90	90.90	89.43	0.90
ResNet50	72.02	0	100	83.74	0.66
VGG19	73.81	6	100	84.61	0.83
DenseNet121	87.50	79.00	91.00	91.29	0.90
Majority class	72.02	0	100	83.73	0.50

### Explainability analysis (XAI)

**Figure 4:**
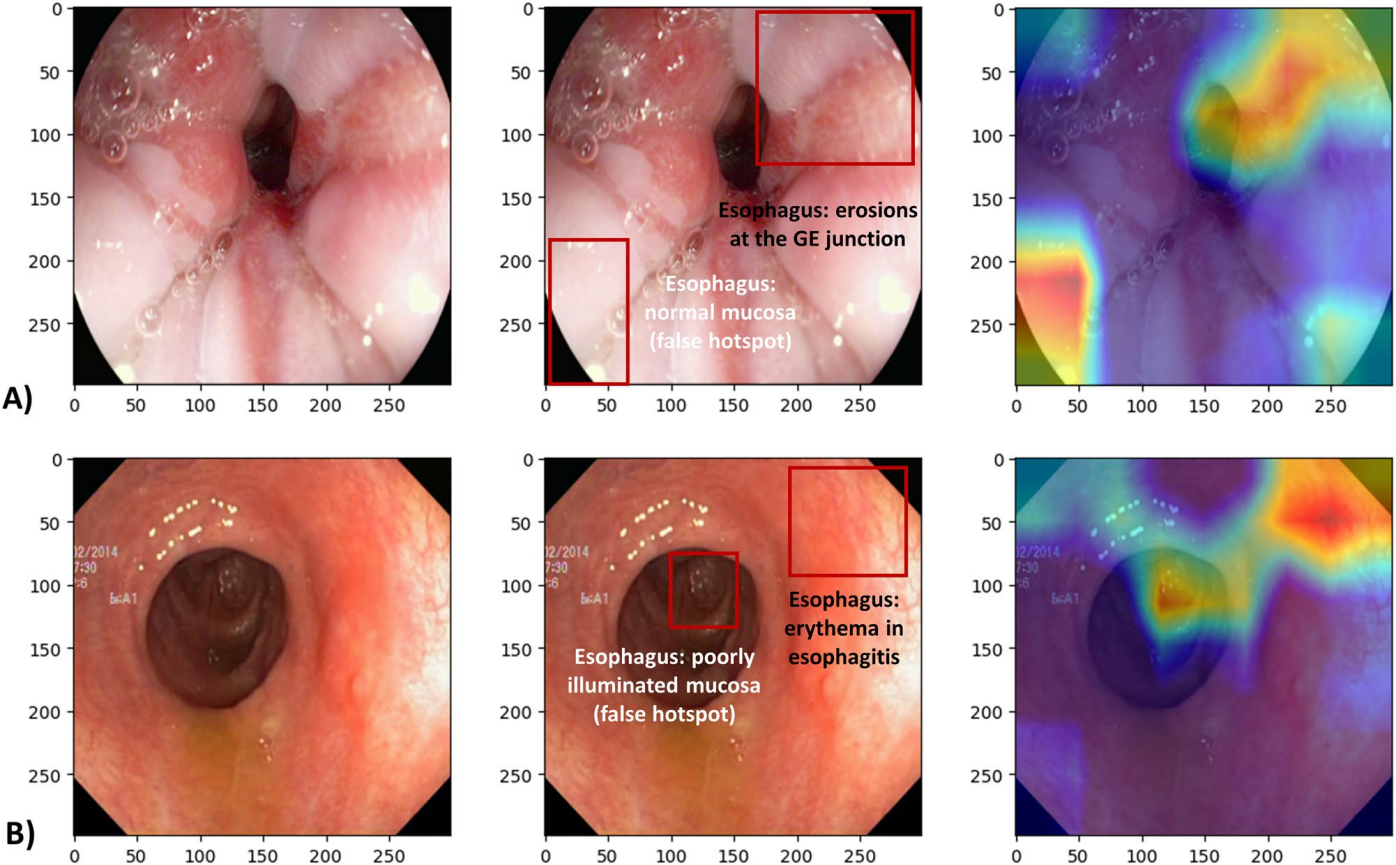
Class activation heatmaps alongside the original endoscopic images. (**A**) Test image (esophagitis) negative for ulcerative colitis, and (**B**) Test image positive for ulcerative colitis. Red color indicates the greatest level of activation. Images included are courtesy of the Center for Open Science and carry a CC-By Attribution 4.0 International license: https://osf.io/mh9sj/.

**Figure 5:**
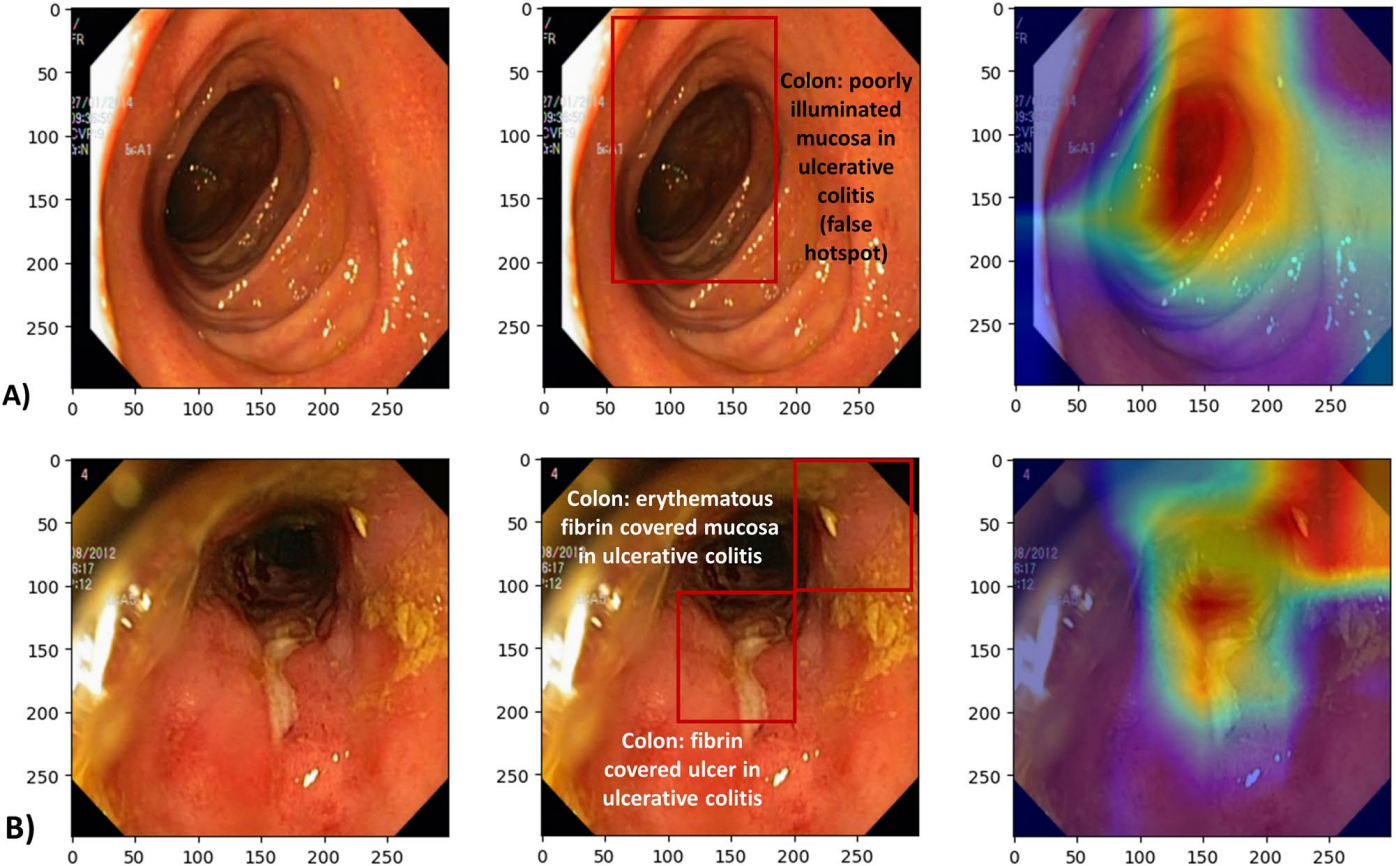
Class activation heatmaps alongside the original endoscopic image for (**A**) Test image with inactive to mild ulcerative colitis (Mayo 0–1), and (**B**) Test image positive for moderate to severe ulcerative colitis (Mayo 2–3). Red color indicates the greatest level of activation. Images included are courtesy of the Center for Open Science and carry a CC-By Attribution 4.0 International license: https://osf.io/mh9sj/.

Gradient-weighted class activation heatmaps for each of the two classification tasks, using DenseNet121 architecture, are shown in Fig. 4 (diagnosis objective) and Fig. 5 (grading objective). The images shown are examples where the model predictions were correct, one for each class (positive or negative). The color scale is from red to orange to blue where red indicates the strongest activation and blue weaker activation.

We note that there can be ambiguity in the heat map indications, compared to the expert analysis. For instance, in Fig. 5B, the heatmaps correctly show the model was activated by fibrin covered ulceration. However, in Fig. 5A activation occurs in the most poorly illuminated portion of the image, thus indicating the model is not using the same information that a human would use to make the classification.

## Discussion

We were able to achieve moderate to good performance in mild vs. moderate-to-severe UC on a relatively small public dataset of endoscopy images. This is remarkable given that images having global (image level) labels (Mayo endoscopic subscores), typically require larger datasets to perform well. By approaching the problem as a binary classification problem, the large differences in bowel wall texture seen between inactive/mild and severe cases might have been easier for the model to distinguish.

We were also able to achieve a high accuracy at distinguishing non-ulcerative colitis endoscopic pathologies from ulcerative colitis. However, it should be noted that this problem and the set of endoscopic pathologies modelled do not represent a major clinical challenge. For example, the dataset included Barret’s esophagus and esophagitis which are found upon endoscopic examination of the upper gastrointestinal tract (i.e., at an esophagogastroduodenoscopy instead of a colonoscopy—examination of the lower gastrointestinal tract). Therefore, for future studies a more appropriate comparator would be lower gastrointestinal tract pathologies at colonoscopies such as diverticulosis, diverticulitis, microscopic colitis, infectious colitis, or pseudomembranous colitis.

Transfer learning with ImageNet weights is a relatively common approach which has shown success in many medical imaging domains^35^. Particularly for smaller datasets, the pre-learned weights on lower layers augments model training, as they are more general features, while the upper layer weights need to become specific to the training task. In this study, end-to-end training was able to achieve good results, but if not, freezing of the lower layers (known as ‘fine-tuning’) could be considered.

Several other studies have investigated automated UC grading with endoscopic images/videos, and have found similar, promising results. Takenaka et al. (2020) deployed an Inceptionv3 network on 40,000 + images from 2012 patients^21^. They achieved 0.945 AUC for predicting endoscopic remission, although they used a total UCEIS score of 0 to indicate remission. Yao et al. (2021) developed an automated video analysis system for grading UC^36^. Their approach differed in that they predicted global whole-video Mayo subscore based on proportions of individual still images exceeding a given Mayo score, by using a template matching grid search algorithm. In high-resolution videos they were able to achieve a classification accuracy of 78%, and 83.7% in a lower resolution test set, although agreement between CNN and humans was not high. Stidham et al. (2019) deployed an InceptionV3 model on 16,514 images from 3082 patients, to predict remission (Mayo 0/1 vs. May 2/3), achieving an impressive AUROC of 0.966^19^. However few studies have investigated or been successful as multi-class classification using each individual Mayo score as a class. Given a large enough dataset, this should be explored.

Additionally, few prior approaches to automated UC grading have addressed explainability (explainable artificial intelligence = XAI), which will be problematic when it comes time to deploy AI models in clinical systems in order to garner physician trust. We attempted to improve explainability of our model by showing representative images of the two classes (positive and negative) along with class activation heatmaps. These heatmaps allow for some speculation as to what patterns the model is identifying to make its prediction. In comparison with the gastroenterologist annotations, we can see that sometimes the heatmaps are identifying clinically representative information, and sometimes they are not. Particularly in Fig. 5B, the heatmaps do suggest the model has learned to recognize fibrin, which is consistent with ulcerative colitis pathology.

As a consequence, we conclude that heatmaps are a good start but remain one of the weaker XAI methods, as they are not semantically driven and can only provide low-level, post-hoc explanations^37^. Other methods such as gradient-based saliency maps, Class Activation Mapping, and Excitation Backpropagation can all be considered in future work^38,39^.

A limitation of this dataset was class imbalance amongst more moderate/severe cases. While we performed stratified splitting, further measures could be taken such as class weighting methods. Ultimately the best solution would be to enrich the dataset. Furthermore, still images present a challenge for Mayo subscore classification, as friability and bleeding may be more difficult to identify in still images than in video. This may have further reduced the true accuracy of the labels provided in the dataset.

Also, two different endoscopes were used, a Pentax (Pentax, Tokyo, Japan) gastroscope for upper endoscopic exams, and an Olympus (Olympus, Tokyo, Japan) colonoscope for the lower, presenting a systematic bias (only for training objective 1 which included both upper and lower pathologies). We consider it unlikely that the model learned to distinguish image features of the scopes themselves based on visual inspection of heatmaps, and equal performance of the model to distinguish ulcerative colitis from upper pathologies, and from polyps, the primary lower pathology. Nonetheless, future work could consider addressing such a bias by performing uniform cropping to remove image outlines, one obvious image feature that could be specific to the endoscope.

In future research we will work with larger and more clinically diverse datasets, and also supplement the feature set with hand-crafted and texture descriptors, for example Color and Edge Directivity Descriptors (CEDD), GLCM, Tamura, or ColorLayout. Such features could be combined with the CNN features by feature fusion at the dense layer (in ‘in vivo’ models real clinical non-image data could also be added in this step). One such approach found good results by using a red density algorithm (red channel) to correlate with endoscopic and histologic disease^40^.

## Data Availability

The data that support the findings of this study are available from the OSF repository, originally published by Borgli et al.^25^.

## Code availability

The Python and MATLAB source code for this project is available upon reasonable request. Python code was compiled in Google Colab. The images were contained in a zip file in the same Google Drive directory as the hosted code.
